# Mechanism of Action of Natural Compounds in Peripheral Multiorgan Dysfunction and Hippocampal Neuroinflammation Induced by Sepsis

**DOI:** 10.3390/antiox12030635

**Published:** 2023-03-03

**Authors:** Ramona D’Amico, Mario Tomasello, Daniela Impellizzeri, Marika Cordaro, Rosalba Siracusa, Livia Interdonato, Ali Saber Abdelhameed, Roberta Fusco, Vittorio Calabrese, Salvatore Cuzzocrea, Rosanna Di Paola

**Affiliations:** 1 Department of Chemical, Biological, Pharmaceutical and Environmental Sciences, University of Messina, Viale Ferdinando Stagno D’Alcontres, n 31, 98166 Messina, Italy; 2 Department of Biomedical and Biotechnological Sciences, University of Catania, 95124 Catania, Italy; 3 Department of Biomedical, Dental and Morphological and Functional Imaging, University of Messina, Via Consolare Valeria, 98125 Messina, Italy; 4 Department of Pharmaceutical Chemistry, College of Pharmacy, King Saud University, Riyadh 14451, Saudi Arabia; 5 Department of Vererinary Sciences, University of Messina, Viale Annunziata, 98168 Messina, Italy

**Keywords:** cecal ligation and perforation, *Coriolus versicolor*, pathway, inflammation, neurodegenerative disorders

## Abstract

Bacterial sepsis induces the production of excessive pro-inflammatory cytokines and oxidative stress, resulting in tissue injury and hyperinflammation. Patients recovering from sepsis have increased rates of central nervous system (CNS) morbidities, which are linked to long-term cognitive impairment, such as neurodegenerative pathologies. This paper focuses on the tissue injury and hyperinflammation observed in the acute phase of sepsis and on the development of long-term neuroinflammation associated with septicemia. Here we evaluate the effects of Coriolus versicolor administration as a novel approach to treat polymicrobial sepsis. Rats underwent cecal ligation and perforation (CLP), and Coriolus versicolor (200 mg/kg in saline) was administered daily by gavage. Survival was monitored, and tissues from vital organs that easily succumb to infection were harvested after 72 h to evaluate the histological changes. Twenty-eight days after CLP, behavioral analyses were performed, and serum and brain (hippocampus) samples were harvested at four weeks from surgery. Coriolus versicolor increased survival and reduced acute tissue injury. Indeed, it reduced the release of pro-inflammatory cytokines in the bloodstream, leading to a reduced chronic inflammation. In the hippocampus, Coriolus versicolor administration restored tight junction expressions, reduce cytokines accumulation and glia activation. It also reduced toll-like receptor 4 (TLR4) and neuronal nitric oxide synthase (nNOS) and the NLR family pyrin domain containing 3 (NLRP3) inflammasome components expression. Coriolus versicolor showed antioxidant activities, restoring glutathione (GSH) levels and catalase and superoxide dismutase (SOD) activities and reducing lipid peroxidation, nitrite and reactive oxygen species (ROS) levels. Importantly, Coriolus versicolor reduced amyloid precursor protein (APP), phosphorylated-Tau (p-Tau), pathologically phosphorylated tau (PHF1), phosphorylated tau (Ser202 and Thr205) (AT8), interferon-induced transmembrane protein 3 (IFITM3) expression, and β-amyloid accumulation induced by CLP. Indeed, Coriolus versicolor restored synaptic dysfunction and behavioral alterations. This research shows the effects of Coriolus versicolor administration on the long-term development of neuroinflammation and brain dysfunction induced by sepsis. Overall, our results demonstrated that Coriolus versicolor administration was able to counteract the degenerative process triggered by sepsis.

## 1. Introduction

Sepsis is described as a life-threatening organ dysfunction induced by dysregulated host answers to infection [1]. It is among the top disorders for the most expensive hospital stays worldwide. 

Inflammatory mediators and oxidative stress have a key role in the development and management of sepsis. Clinical evidence shows that following bacterial peritonitis, a huge intraperitoneal cytokine response occurs, with high levels of pro-inflammatory cytokines and oxidative mediators [2]. This mediator response is responsible for the uncontrolled activation of the systemic inflammation via the MD2–toll-like receptor 4 (TLR4). It causes the production of the inflammatory cytokines (interleukin (IL)-6, tumor necrosis factor (TNF)-α, IL-1β) responsible for hyper-inflammation [3,4]. Up to 80% of patients affected by sepsis potentially develop irreversible cerebral dysfunction [5,6], caused by systemic inflammation [7]. Previous researchers have underlined many long-lasting results following sepsis recovery, including brain disorders [8]. In particular, animals subjected to cecal ligation and perforation (CLP) showed important difficulties in performing behavioural tests [9]. In septic patients, persistent difficulties in brain functional activities and quality of life have been observed [10], which may be related to long-lasting difficulties in cognitive function associated with executive and memory tasks [11]. This impairment in brain activity has been proposed to result from ischemic or neurodegenerative pathways activated by systemic inflammation [12]. Peripherally released cytokines can reach the central nervous system (CNS), as sepsis may induce the disruption of the blood–brain barrier (BBB) [13,14]. Local release of cytokines may induce astrocyte/microglia activation, which in turn produces reactive oxygen species (ROS) and pro-inflammatory mediators [15,16]. However, aspects of the molecular cascades relating systemic inflammation to neuroinflammation and brain dysfunction still need to be clarified. A detailed understanding of these pathways may provide important information useful to enact new strategies to counteract sepsis co-morbidities. Novel knowledge on the molecular mechanisms connecting systemic inflammation to brain diseases may also be helpful to understand the onset of neuroinflammation itself [17]. Neurodegenerative progressions may evolve over the years, and the diagnosis is mostly performed only in the late stages of the disease, when a significant neuronal loss impairs the brain functions. Neuroinflammation is characterized by gradual neuronal death accompanying by the accumulation of aberrant, misfolded forms of proteins or peptides with neurotoxic activity. Although the complete molecular steps of these events need to be clarified, the occurrence of reduced tight junctions and augmented Aβ accumulation in amyloid plaques in the hippocampus, together with the previous data mentioned above, are highly indicative of neurotoxic processes that could represent the early molecular processes of neurodegenerative cascades during activation. Therefore, we propose that compounds with antioxidant, anti-inflammatory and neuroprotective activities may be a good approach for the prevention of sepsis-related neurodegenerative diseases. 

Mushrooms contain many bioactive compounds that suggest their use for human health and the prevention of diseases related to CNS. *Coriolus versicolor* is a well characterized mushroom that has shown important neuroprotective effects [18]. Previous studies displayed the anti-inflammatory, antioxidant, antibacterial, anticancer and immunomodulatory properties [19,20,21,22]. In particular, it has been suggested that *Coriolus versicolor* increases the cellular redox potential, inducing the vitagene defense system, including lipoxin A4, thioredoxin and heat shock protein 70 [23]. Based on these findings, this paper aims to evaluate the role of *Coriolus versicolor* administration in acute inflammation and long-term development of neuroinflammation and brain dysfunction triggered by chronic inflammation, induced by polymicrobial sepsis.

## 2. Materials and Methods

### 2.1. Animals

Male Wistar rats (250–280 g, 6–8 weeks old) (Envigo, Milan, Italy) were housed in a controlled environment and provided with standard chow (Teklad, Milan, Italy) and water. The University of Messina Review Board for Animal Care (OPBA) approved the study that followed the Italian D.Lgs 2014/26 and EU regulations (EU Directive 2010/63).

### 2.2. Preparation of Coriolus versicolor Extract

*Coriolus versicolor* biomasses, containing mycelium and primordia, generously donated by Mycology Research Laboratories Ltd. (MRL, Luton, UK), as a commercially available product, were used for the research. The optimal dose (200 mg/kg) was chosen based on the dose used in human trials with cancer or HPV patients (3 g/day), a regimen also verified by rat investigations [18]. 

The characterization of the two fungi was performed by Chromatography-Orbitrap-Mass Spectrometry (LC-Orbitrap-MS) and by Gas Chromatography-Tandem Mass Spectrometry (GC-MS/MS). 

### 2.3. CLP Induction

Animals were subjected to sham-surgery or CLP, as already described [24]. Rats were anesthetized (Sevorane 2%), and a midline laparotomy was performed to expose the cecum. This was tightly ligated below the ileocecal valve, perforated and squeezed to extrude a small amount of feces. The cecum was then returned to the peritoneal cavity, and the abdomen was sutured. In the animals assigned to the sham group (Control), the laparotomy and cecal exposure were performed, but no further manipulations were applied. After surgery, 50 mL/kg of saline was subcutaneously injected with 30 mg/kg ceftriaxone and 25 mg/kg clindamycin every 6 h for 3 days. These drugs were administered to both sham and CLP groups. Immediately after surgery, animals were monitored for twenty-eight days for survival and daily activity.

### 2.4. Experimental Groups

Animals were randomly divided as following:

Control: rats were subjected to the surgical procedures, but no ligation or perforation were performed, and vehicle (saline) was administered daily by gavage; 

Control + *Coriolus versicolor*: rats were subjected to the surgical procedures, but no ligation or perforation were performed and *Coriolus versicolor* (200 mg/kg dissolved in saline) was administered daily by gavage for 28 days;

CLP: rats were subjected to the procedure previously described and vehicle was administered daily by gavage; 

CLP+ *Coriolus versicolor*: rats were subjected to the procedure previously described, and *Coriolus versicolor* (200 mg/kg dissolved in saline) was administered daily by gavage for 28 days.

The dose of *Coriolus versicolor* was chosen based on previous studies [23]. A “pilot” evaluation, using n = 10 animals per group, was performed in order to investigate the effect of Coriolus versicolor administration on organ dysfunction and tissue damage induced by sepsis. In accordance with what was previously described in the literature [25], a high mortality rate was observed at 72 h. Therefore, in the second part of the work, due to the elevated mortality observed in the pilot study, we employed n = 50 animals for each group. G power software (3.1 version) was employed to determine the minimum number of animals required to obtain a statistically significant outcome (effect size f = 0.25; α err prob = 0.05; power (1-β err prob) = 0.95). After twenty-eight days from the surgery, the behavioral tasks were performed, and the animals were sacrificed (euthanasia: Sevorane overexposure). 

### 2.5. Histological Analysis

Kidney, liver, lungs and gut from all experimental groups were harvested, fixed in buffered formaldehyde solution (10% in PBS), dehydrated and embedded in Paraplast [26]. Tissue slides were stained with H & E and evaluated using a Leica DM6 microscope (Leica Microsystems SpA, Milan, Italy). Histopathologic scores were evaluated following the methodology adapted by a board-certified pathologist [27]. 

### 2.6. Behavioral Task

#### 2.6.1. Morris Water Maze (MWM)

Spatial learning and memory consolidation were evaluated by MWM test [28]. To conduct the experiment, a circular water container measuring 60 cm in height and 152 cm in diameter was filled with water maintained at 23 °C to a depth of 30 cm. An escape platform measuring 10 cm in diameter and located 2 cm below the water surface was fixed in a quadrant of the tank throughout the experiment. A white curtain was draped around the tank, and four types of black paper with different shapes were attached to the inside of the curtain. Each animal was subjected to a daily trial session for four days, followed by a probe trial 24 h after the last training session. The time spent in the target quadrant and the percentage of distance covered were measured. The experimental setup was obtained from Ugo Basile in Milan, Italy.

#### 2.6.2. Elevated Plus Maze (EPM)

Memory-related processes were evaluated by the EPM test performed as already described [29]. The animals were placed individually at the open end of the maze’s open arm, facing towards the opposite end of the maze, using equipment from Ugo Basile in Milan, Italy. The time taken by the animal to move from the open arm to the closed arm was recorded as the initial acquisition latency (IAL). After recording the IAL, the animal was permitted to explore the maze for 20 s before being returned to its home cage. If the animal failed to enter the enclosed arms within 90 s, it was pushed into one of the enclosed arms, and the IAL was recorded as 90 s. On the 28th day following CLP, the rat’s memory retention was evaluated by placing it in an open arm, and the retention transfer latency (RTL) was noted during the re-test session.

#### 2.6.3. Novel Object Recognition (NOR)

Changes in cognitive function were assessed by NOR [30]. Recognition index (RI) was identified as the time spent exploring the novel object and was calculated by dividing the time spent investigating the novel object (TN) by the time spent exploring the TN and a familiar object (TF), [RI = TN/(TN + TF)]. 

#### 2.6.4. Open Field Test

An open field test was executed to check the mental condition of an animal via locating the rats in a bright light-illuminated box noticeably bigger than the home confine [31]. The apparatus consisted of a large square box of 100 cm length × breadth with 40 cm height made up of plywood walls (Ugo Basile, Milan, Italy). The number of nurturing and squares in a 5 min break was noted and tabulated, and it was regarded as a locomotion activity parameter [32].

### 2.7. BBB Permeability Studies

BBB permeability was measured in terms of sodium fluorescein [33]. Rats were intravenously injected with 100 mg/mL of sodium fluorescein (350 Da). Thereafter, they were perfused with PBS, and brain tissues were collected. The hippocampi were weighed and homogenized. Proteins were precipitated with 20% trichloroacetic acid, and samples were centrifuged. The fluorescent intensity was red at 480–538 nm [34]. 

### 2.8. Immunohistochemical Analysis

Brain samples were collected, processed, and embedded in paraffin [35]. Sections of 7 μm thickness were prepared. Immunohistochemical localization was performed as already described [36]. After deparaffinization, endogenous peroxidase was quenched with 0.3% (*v*/*v*) hydrogen peroxide in 60% (*v*/*v*) water for 30 min. The slides were permeabilized with 0.1% (*w*/*v*) Triton X-100 in PBS for 20 min [37]. Tissue sections were incubated in 2% (*v*/*v*) normal goat serum in PBS to block non-specific binding. Sequential incubation for 15 min with avidin and biotin (Vector Laboratories, Burlingame, CA, USA) was performed to block, respectively, endogenous avidin or biotin binding sites [38]. The sections were incubated overnight with primary antibodies (Table 1).

All sections were washed with PBS and then treated as previously reported [39] and incubated with secondary antibody. Specific labelling was identified with a biotin-conjugated goat anti-rabbit IgG and avidin–biotin peroxidase complex [40]. Stained sections were observed using a Leica DM6 microscope (Leica Microsystems SpA, Milan, Italy) following a typical procedure [41].

The photographs obtained (n = 5 photos from five slides for each sample) were collected from all animals in each experimental group. The digital images were opened in ImageJ, followed by deconvolution using the color deconvolution plug-in. When the IHC profiler plug-in is selected, it automatically plots a histogram profile of the deconvoluted DAB image, and a corresponding scoring log is displayed. The histogram profile corresponds to the positive pixel intensity value obtained from the computer program. All immunohistochemical analyses were carried out by two observers blinded to the treatment.

### 2.9. Western Blot Analysis

Western blot analyses were performed as previously described [42]. Tissues were lysed with RIPA buffer containing a cocktail of protease inhibitors and phosphatase inhibitors. Lysate was centrifuged at 10,000× *g* at 4 °C for 15 min, and the supernatant was collected. The total protein concentration was determined using the Bradford assay (Bio-rad Laboratories). Equal amounts of protein were loaded onto SDS-PAGE gel and then transferred onto a PVFD membrane. After blocking with 5% skimmed milk, filters were probed with one of the primary antibodies (Table 1) mixed in a 5% *w*/*v* nonfat dried milk solution and incubated at 4 °C overnight. Blots were incubated with a peroxidase-conjugated bovine anti-mouse IgG secondary antibody or a peroxidase conjugated goat anti-rabbit IgG for 1 h at room temperature [43]. Membranes were also incubated with an antibody against β-actin (Santa Cruz Biotechnology, Dallas, TX, USA) to verify that the amounts of protein were equal. Signals were detected with an enhanced chemiluminescence detection system reagent (Super-Signal West Pico Chemiluminescent Substrate, Pierce). The relative expression of the protein bands was quantified by densitometry with Bio-Rad ChemiDoc XRS software and standardized to β-actin levels. Images of blot signals were imported to an analysis software (Image Quant TL, v2003) [44].

### 2.10. Cytokines Measurement

Serum and Hippocampal levels of IL 6, TNF-α, IL-1β, IL 18 and β-amyloid were determined using an ELISA kit (Diaclone Research, Biosource Europe, USCN life Sciences; Invitrogen, Milan, Italy). Briefly, tissues were homogenized in 1 mL PBS with 10 μL protease inhibitor at low speed. The samples were centrifuged at 14,000× *g* at 4 °C for 15 min; supernatants were employed, using respective ELISA kits according to the manufacturer’s protocol, and analyzed using a microplate reader [29,45].

### 2.11. Biochemical Analysis

Biochemical analyses were conducted on the hippocampus:

To evaluate superoxide dismutase (SOD) activity, samples were homogenized in Tris buffer (pH 8.2) and centrifuged at 10,000× *g*. TritonX-100 was added; samples were incubated at 4–8 °C for 20 min and then centrifuged at 8000× *g*. The absorbance was measured at 420 nm. [46]. To evaluate catalase (CAT) activity, tissues were homogenized in phosphate buffer at 800 g, then hydrogen peroxide was added and absorbance was measured for 0–10 min at 240 min [47]. To evaluate glutathione (GSH) levels, trichloroacetic acid solution was added to homogenized samples (0.2 M phosphate buffer (pH 7.6)). Then, the mixture was centrifuged at 3900× *g*. 5,5′-dithiobis-(2-nitrobenzoic acid) was added, and samples were incubated at room temperature for 5 min and the absorbance was measured at 412 nm [48]. To evaluate nitrite levels, Griess reagent was added to homogenized samples. The absorbance was measured at 548 nm [28]. To evaluate lipid peroxidation, thiobarbituric acid-reactant substance evaluation was performed [49]. Samples were homogenized in Hank’s balanced salt solution at 2000 g. Pellets were incubated in a solution containing sodium dodecyl sulfate, acetic acid, thiobarbituric acid, and water for 1 h at 95 °C. After cooling, water, n-butanol, and pyridine were added, and the mixture was centrifuged at 2000× *g*. The absorbance was measured at 532 nm. To evaluate the production of ROS in the brain, dichlorofluorescein diacetate (DCFH-DA) was added to homogenized samples. The conversion of non-fluorescent DCFH-DA to the highly fluorescent compound 20,70-dichlorofluorescein (DCF) by esterase activity was used to monitor the presence of ROS due to the oxidative burst in the brain [28]. 

### 2.12. Statistical Evaluation

All values are expressed as mean ± standard error of the mean (SEM) of N = 30 observations. For in vivo studies, N represents the number of animals used. Results were analyzed by Log-rank (Mantel–Cox) test or one-way ANOVA, followed by a Bonferroni post hoc test for multiple comparisons. A *p*-value of less than 0.05 was considered significant. * *p* < 0.05 vs. Control, # *p* < 0.05 vs. CLP, ** *p* < 0.01 vs. Control, ## *p* < 0.01 vs. CLP, *** *p* < 0.001 vs. Control, ### *p* < 0.001 vs. CLP.

## 3. Results

### 3.1. Effects of Coriolus versicolor on Survival after CLP Induction

**Figure 1:**
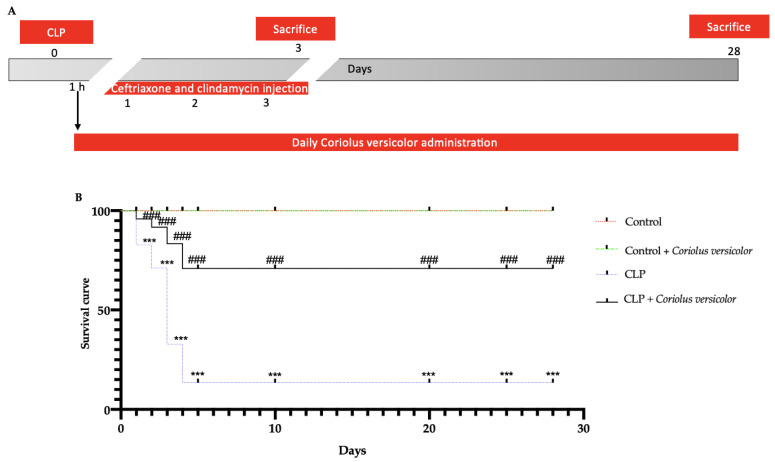
*Coriolus versicolor* reduced CLP-induced mortality: Experimental timeline (**A**), Percent survival (**B**). For these analysis n = 10 animals for each group were employed. Results were analyzed by Log-rank (Mantel-Cox) test. A *p*-value of less than 0.05 was considered significant. *** *p* < 0.001 vs. Control, ### *p* < 0.001 vs. CLP.

Male Wistar rats were subjected to CLP, and *Coriolus versicolor* (200 mg/kg in saline) was administered daily by gavage (Figure 1A). Survival was monitored over 28 days (Figure 1B). No mortality was detected in the Control and Control + *Coriolus versicolor* groups. Animals from the CLP group showed a survival of 82% at day 1, 71% at day 2, 32% at day 3, and 13% at day 4 to day 28 from the surgery. The CLP + *Coriolus versicolor* group showed a survival of 95% at day 1, 91% at day 2, 83% at day 3, and 70% at day 4 to day 28 from the surgery.

### 3.2. Effects of Coriolus versicolor on Organ Damage CLP Induced 

**Figure 2:**
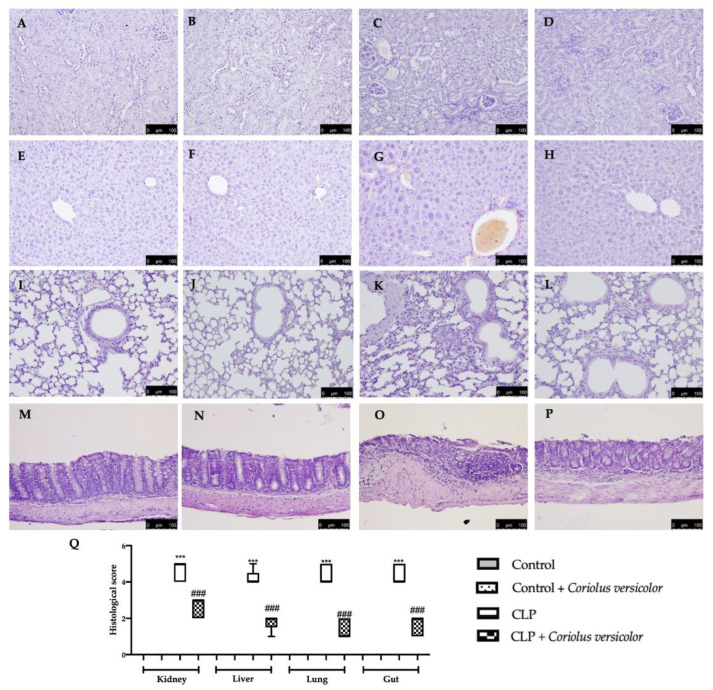
*Coriolus versicolor* reduced histological damage induced by CLP 72 h after surgery. Hematoxylin and eosin staining of: kidney: Control (**A**), Control + *Coriolus versicolor* (**B**), CLP (**C**), CLP + *Coriolus versicolor* (**D**); liver: Control (**E**), Control + *Coriolus versicolor* (**F**), CLP (**G**), CLP + *Coriolus versicolor* (**H**); lungs: Control (**I**), Control + *Coriolus versicolor* (**J**), CLP (**K**), CLP + *Coriolus versicolor* (**L**); gut: Control (**M**), Control + *Coriolus versicolor* (**N**), CLP (**O**), CLP + *Coriolus versicolor* (**P**); histological score (**Q**). *** *p* < 0.001 vs. Control, ### *p* < 0.001 vs. CLP.

The high bacterial load induced by sepsis caused an exaggerated inflammatory response, resulting in organ dysfunction and tissue damage. To test whether *Coriolus versicolor* restored organ damage against sepsis-induced injury, we analyzed tissues from vital organs that easily succumb to infection such as the kidney, liver, lungs, and gut from all experimental groups to study histopathological changes. All the tissues from different experimental groups were harvested after 72 h. This is because the CLP group showed a high mortality beyond 72 h, while the CLP + *Coriolus versicolor* group lived longer. No histological damage was detected in the Control groups (Figure 2A,B,E,F,I,J,M,N). Tissues from the CLP group showed microthrombi and congestion in the kidney (Figure 2C,Q), liver (Figure 2G,Q), and lungs (Figure 2K,Q) and increased necrosis of villi in gut (Figure 2O,Q), as compared to controls. Treatment with *Coriolus versicolor* reversed these changes in all organs studied (Figure 2D,H,I,P).

### 3.3. Effects of Coriolus versicolor on Long-Lasting Elevation of Serum Cytokines CLP Induced

**Figure 3:**
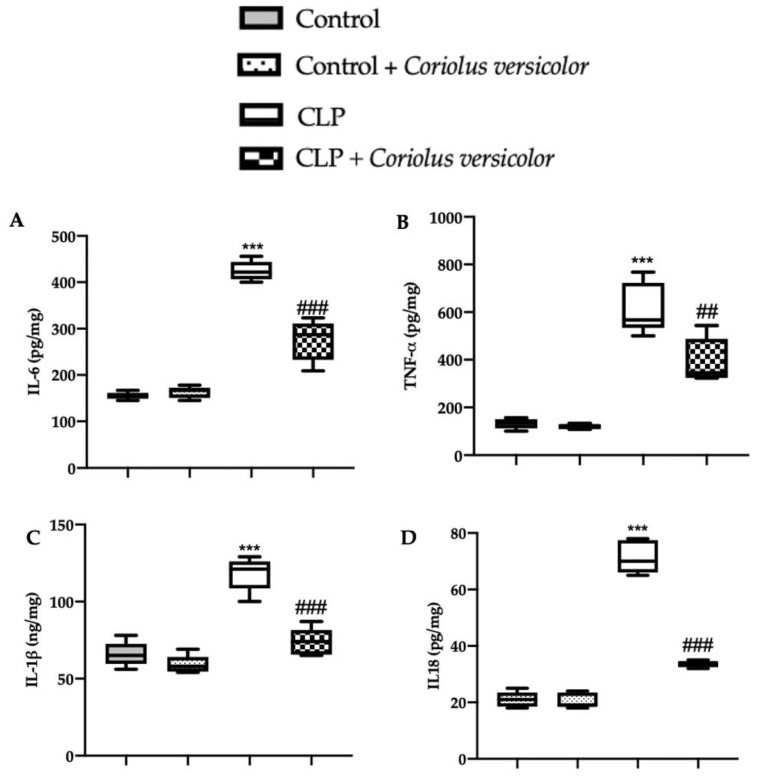
*Coriolus versicolor* reduced serum cytokines induced by CLP 28 days after surgery. Levels of serum interleukin (IL)6 (**A**), tumor necrosis factor (TNF)-α (**B**), IL-1β (**C**), IL18 (**D**). *** *p* < 0.001 vs. Control, ## *p <* 0.01 vs. CLP, ### *p* < 0.001 vs. CLP.

In order to evaluate the anti-inflammatory effect of *Coriolus versicolor* administration, serum cytokine levels were assessed. No differences in IL6 (Figure 3A), TNF-α (Figure 3B), IL-1β (Figure 3C), or IL18 (Figure 3D) levels between Control and Control + *Coriolus versicolor* were determined. The CLP group showed increased expression of cytokines, as compared to the controls. The CLP + *Coriolus versicolor* group showed reduced levels of IL6 (Figure 3A), TNF-α (Figure 3B), IL-1β (Figure 3C), and IL18 (Figure 3D) in serum, as compared to the CLP group. 

### 3.4. Effects of Coriolus versicolor on Long-Lasting Reduction of Hippocampal Tight Junctions CLP Induced

**Figure 4:**
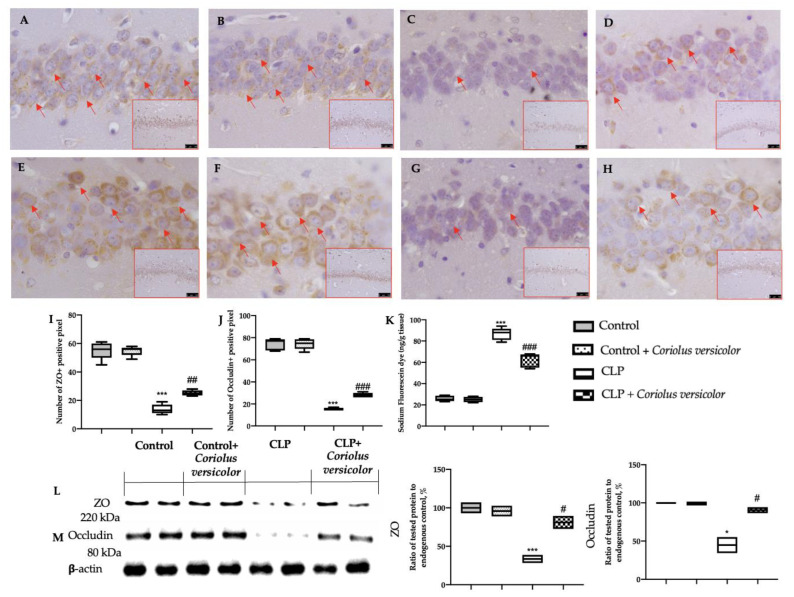
*Coriolus versicolor* reduced tight junctions alterations in the hippocampus 28 days after CLP. Immunohistochemical analysis of zona occludens (ZO): Control (**A**), Control + *Coriolus versicolor* (**B**), CLP (**C**), CLP + *Coriolus versicolor* (**D**), immunohistochemical analysis of occludin: Control (**E**), Control + *Coriolus versicolor* (**F**), CLP (**G**), CLP + *Coriolus versicolor* (**H**). Graphical quantification of ZO expression (**I**); graphical quantification of ZO expression (**J**). Sodium fluorescein extravasation (**K**). Western blot analysis of ZO (**L**), occludin (**M**). * *p <* 0.05 vs. Control, # *p <* 0.05 vs. CLP, ## *p* < 0.01 vs. CLP, *** *p* < 0.001 vs. control, ### *p* < 0.001 vs. CLP.

Basal levels of ZO and occludin expression were detected in Control (Figure 4A,I) and Control + *Coriolus versicolor* (Figure 4E,J) groups, and no statistical differences were found between them (Figure 4I,J). ZO and occludin expression were found to be strongly reduced in the CLP group (Figure 4C,G), as compared to the controls, while *Coriolus versicolor* administration partially restored their levels (Figure 4D,H). The analysis of the BBB permeability, conducted by the sodium fluorescein dye extravasation, showed increased permeability in the hippocampi of the CLP group, as compared to the control groups. *Coriolus versicolor* administration significantly reduced the dye extravasation (Figure 4K), as compared to the CLP group. Western blot analysis also confirmed these data. ZO (Figure 4L) and occludin (Figure 4M) levels decreased in the CLP group, as compared to the controls, while *Coriolus versicolor* administration increased their expressions. 

### 3.5. Effects of Coriolus versicolor on Long-Lasting Elevation of Hippocampal Cytokines CLP Induced

**Figure 5:**
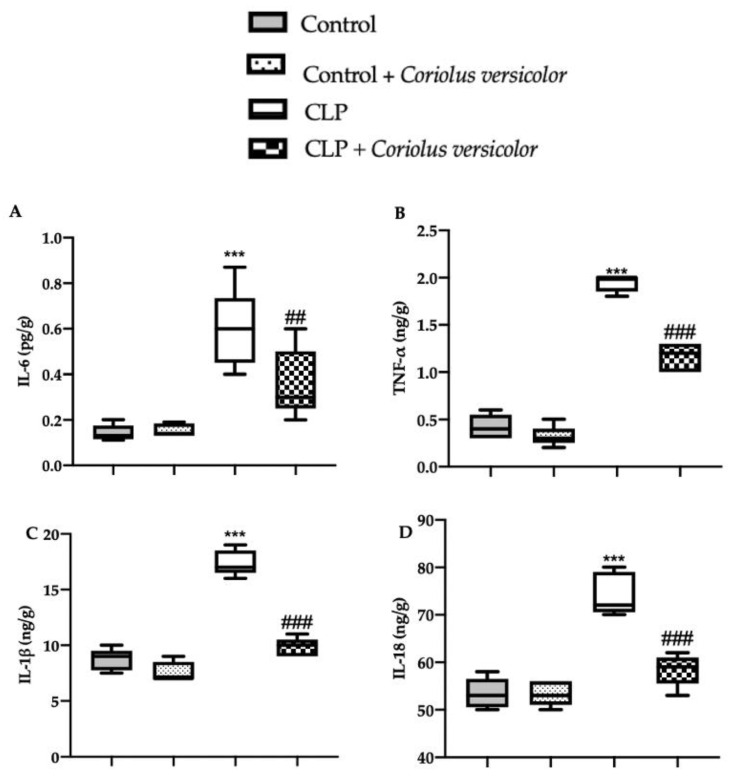
*Coriolus versicolor* reduced hippocampal cytokines induced by CLP 28 days after surgery. Levels of hippocampal interleukin (IL)6 (**A**), tumor necrosis factor (TNF)-α (**B**), IL-1β (**C**), IL18 (**D**). ## *p* < 0.01 vs. CLP, *** *p* < 0.001 vs. control, ### *p* < 0.001 vs. CLP.

In order to investigate the neuroprotective effects of *Coriolus versicolor* administration, hippocampal cytokine levels were assessed. No differences in IL6 (Figure 5A), TNF-α (Figure 5B), IL-1β (Figure 5C), and IL18 (Figure 5D) levels between Control and Control + *Coriolus versicolor* were determined. The CLP group showed increased expression of cytokines, as compared to the controls. The CLP + *Coriolus versicolor* group showed reduced levels of IL6 (Figure 5A), TNF-α (Figure 5B), IL-1β (Figure 5C), and IL18 (Figure 5D) in the hippocampus, as compared to CLP. 

### 3.6. Effects of Coriolus versicolor on Activation of Hippocampal Glial Cell CLP Induced

**Figure 6:**
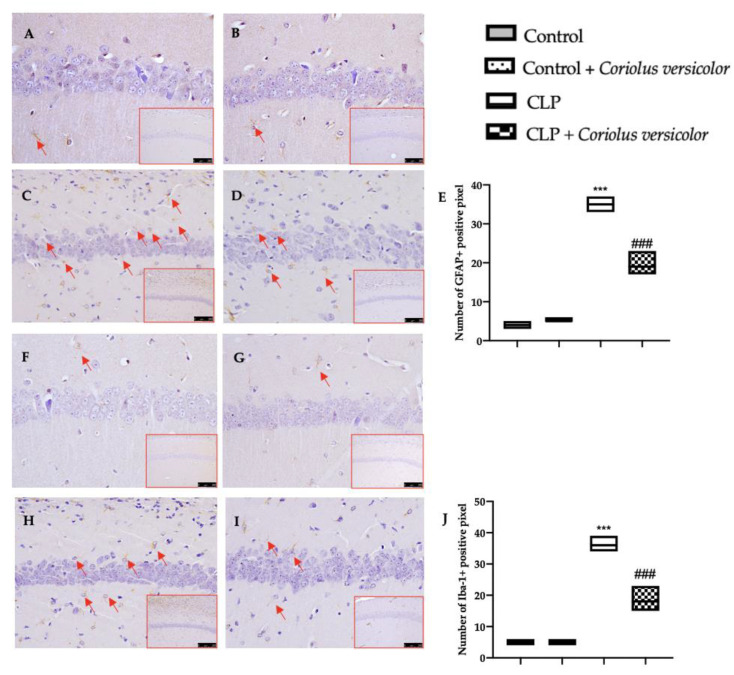
*Coriolus versicolor* reduced glia activation in hippocampus induced by CLP 28 days after surgery. Immunohistochemical analysis of glial fibrillary acidic protein (GFAP): Control (**A**), Control + *Coriolus versicolor* (**B**), CLP (**C**), CLP + *Coriolus versicolor* (**D**), graphical quantification of GFAP expression (**E**); immunohistochemical analysis of Ionized calcium-binding adapter molecule1 (Iba-1): Control (**F**), Control + *Coriolus versicolor* (**G**), CLP (**H**), CLP + *Coriolus versicolor* (**I**), graphical quantification of Iba-1 expression (**J**). *** *p* < 0.001 vs. control, ### *p* < 0.001 vs. CLP.

**Figure 7:**
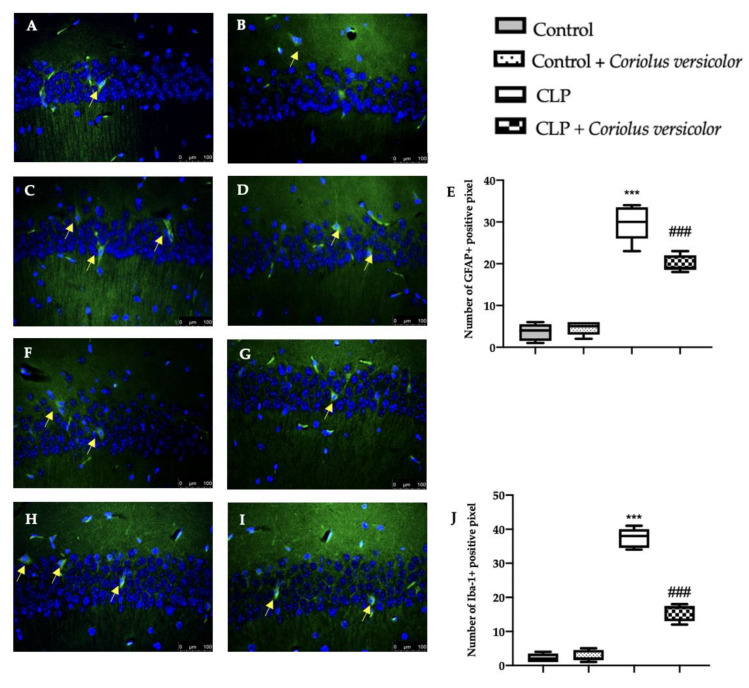
*Coriolus versicolor* reduced glia activation in hippocampus induced by CLP 28 days after surgery. Immunofluorescence analysis of glial fibrillary acidic protein (GFAP): Control (**A**), Control + *Coriolus versicolor* (**B**), CLP (**C**), CLP + *Coriolus versicolor* (**D**), graphical quantification of GFAP expression (**E**); immunofluorescence analysis of Ionized calcium-binding adapter molecule1 (Iba-1): Control (**F**), Control + *Coriolus versicolor* (**G**), CLP (**H**), CLP + *Coriolus versicolor* (**I**), graphical quantification of Iba-1 expression (**J**). *** *p* < 0.001 vs. control, ### *p* < 0.001 vs. CLP.

To investigate the glia activation, immunohistochemical and immunofluorescence analyses were conducted. GFAP and Iba-1 expression were found strongly increased in the CLP group (Figure 6C,H and Figure 7C,H), as compared to the control groups (Figure 6A,B,F,G and Figure 7A,B,F,G). No statistical differences were determined between Control (Figure 6E and Figure 7E) and Control + *Coriolus versicolor* (Figure 6J and Figure 7J) groups. *Coriolus versicolor* decreased both glial fibrillary acidic protein (GFAP) (Figure 6D and Figure 7D) and Ionized calcium-binding adapter molecule1 (Iba-1) (Figure 6I and Figure 7I) expressions, as compared to CLP.

### 3.7. Effects of Coriolus versicolor on Hippocampal Inflammation CLP Induced

**Figure 8:**
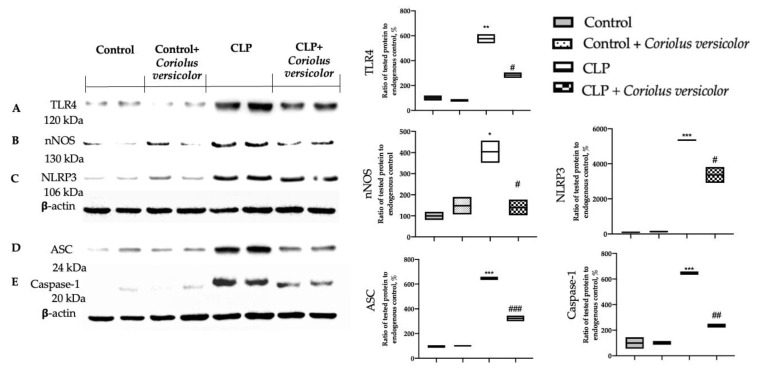
*Coriolus versicolor* reduced hippocampal inflammation induced by CLP 28 days after surgery. Western blot analysis of toll-like receptor 4 (TLR4) (**A**), nitric oxide synthase (nNOS) (**B**), NLR family pyrin domain containing 3 (NLRP3) (**C**), apoptosis-associated speck-like protein containing a CARD (ASC) (**D**), Caspase-1 (**E**) expression. * *p* < 0.05 vs. control, # *p* < 0.05 vs. CLP, ** *p* < 0.01 vs. control, ## *p* < 0.01 vs. CLP, *** *p* < 0.001 vs. control, ### *p* < 0.001 vs. CLP.

**Figure 9:**
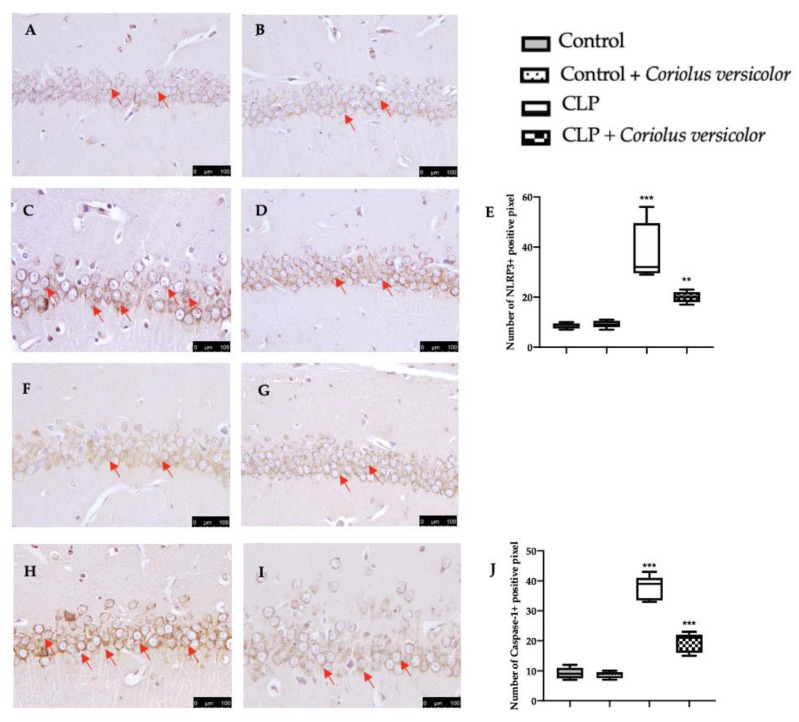
*Coriolus versicolor* reduced NLR family pyrin domain containing 3 (NLRP3) and Caspase-1 expression induced by CLP 28 days after surgery. Immunohistochemical analysis of NLRP3: Control (**A**), Control + *Coriolus versicolor* (**B**), CLP (**C**), CLP + *Coriolus versicolor* (**D**), graphical quantification of NLRP3 expression (**E**), immunohistochemical analysis of Caspase-1: Control (**F**), Control + *Coriolus versicolor* (**G**), CLP (**H**), CLP + *Coriolus versicolor* (**I**), graphical quantification of Caspase-1 expression (**J**). ** *p* < 0.01 vs. Control, *** *p* < 0.001 vs. control.

**Figure 10:**
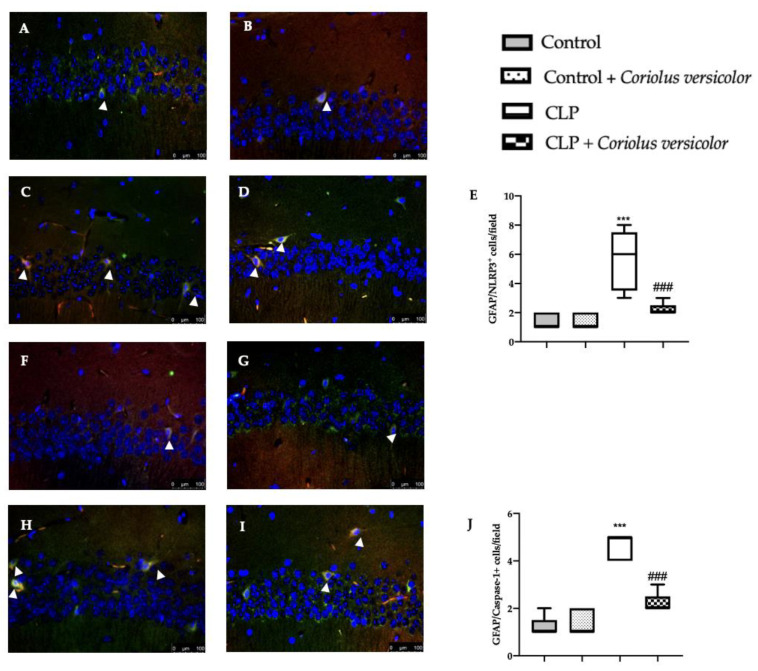
*Coriolus versicolor* reduced glial fibrillary acidic protein (GFAP)/NLR family pyrin domain containing 3 (NLRP3) and GFAP/Caspase-1 colocalization induced by CLP 28 days after surgery. Immunofluorescence analysis of GFAP/NLRP3: Control (**A**), Control + *Coriolus versicolor* (**B)**, CLP (**C**), CLP + *Coriolus versicolor* (**D**), graphical quantification of GFAP/NLRP3 colocalization (**E**), immunofluorescence analysis of NLRP3/Caspase-1: Control (**F**), Control + *Coriolus versicolor* (**G**), CLP (**H**), CLP + *Coriolus versicolor* (**I**), graphical quantification of NLRP3/Caspase-1 colocalization (**J**). *** *p* < 0.001 vs. control, ### *p* < 0.001 vs. CLP.

**Figure 11:**
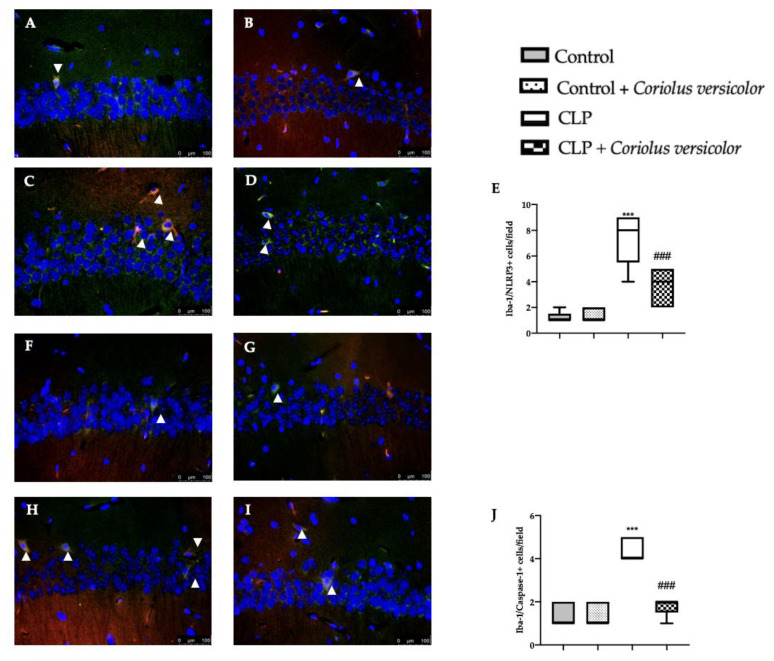
*Coriolus versicolor* reduced Ionized calcium-binding adapter molecule1 (Iba-1)/ NLR family pyrin domain containing 3 (NLRP3) and Iba-1/Caspase-1 colocalization induced by CLP 28 days after surgery. Immunofluorescence analysis of Iba-1/NLRP3: Control (**A**), Control + *Coriolus versicolor* (**B**), CLP (**C**), CLP + *Coriolus versicolor* (**D**), graphical quantification of Iba-1/NLRP3 colocalization (**E**), immunofluorescence analysis of Iba-1/Caspase-1: Control (**F**), Control + *Coriolus versicolor* (**G**), CLP (**H**), CLP + *Coriolus versicolor* (**I**), graphical quantification of Iba-1/Caspase-1 colocalization (**J**). *** *p* < 0.001 vs. control, ### *p* < 0.001 vs. CLP.

To evaluate the neuroinflammation CLP induced, Western blot analyses were conducted. Increased expression of TLR4 (Figure 8A) and nitric oxide synthase (nNOS) (Figure 8B) were found in tissues harvested from the CLP group, as compared to the Control and Control + *Coriolus versicolor* groups. Tissues harvested from CLP + *Coriolus versicolor* group showed reduced TLR4 (Figure 8A) and nNOS (Figure 8B). CLP also increased the expression of the NLR family pyrin domain containing 3 (NLRP3) inflammasome components: NLRP3 (Figure 8C), apoptosis-associated speck-like protein containing a CARD (ASC) (Figure 8D), and Caspase-1 (Figure 8E), as compared to the Control and Control + *Coriolus versicolor* groups. *Coriolus versicolor* administration strongly reduced NLRP3 (Figure 8C), ASC (Figure 8D), and Caspase-1 (Figure 8E) expression, as compared to CLP. Immunohistochemical analysis confirmed the increased expression of NLRP3 and Caspase-1 in the CLP group (Figure 9C, Figure 9D, Figure 9H, and Figure 9J, respectively), as compared to the Control (Figure 9A,F) and Control + *Coriolus versicolor* groups (Figure 9B,G). Well in line with the Western blot results, the CLP + *Coriolus versicolor* group showed reduced NLRP3 (Figure 9D) and Caspase-1 (Figure 9I) expression, as compared to CLP. Indeed, fluorescent-double-staining showed an increased number of GFAP/NLRP3 and GFAP/Caspase-1-positive cells in samples harvested from the CLP group (Figure 10C,E,H,J), as compared to the samples harvested from the Control (Figure 10A,E,F,J) and Control + *Coriolus versicolor* (Figure 10B,E,G,J) groups. CLP + *Coriolus versicolor* group showed a reduced number of GFAP/NLRP3 (Figure 10D,E) and GFAP/Caspase-1 (Figure 10I,J) positive cells. Additionally, the CLP group showed an increased number of Iba-1/NLRP3 (Figure 11C,E) and Iba-1/Caspase-1 (Figure 11H,J) positive cells as compared to controls (Figure 11A,B,E–G,J). 

### 3.8. Effects of Coriolus versicolor on Oxidative Stress CLP Induced

**Figure 12:**
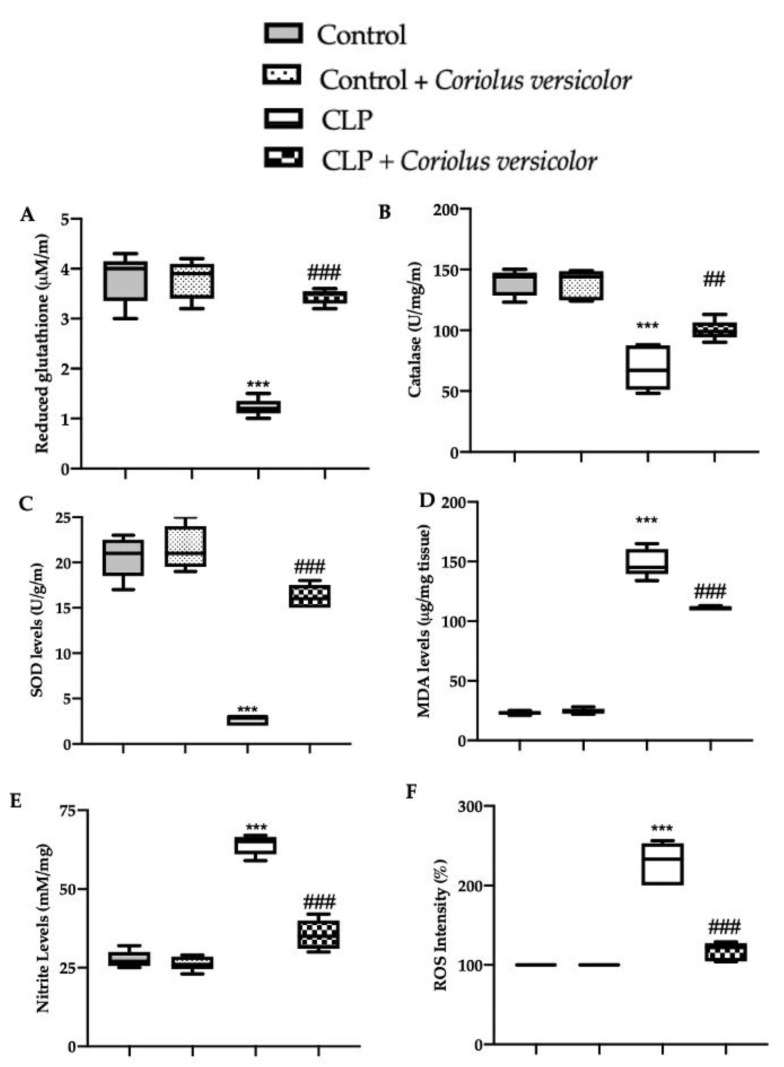
*Coriolus versicolor* reduced hippocampal oxidative stress induced by CLP 28 days after surgery. Glutathione (GSH) levels (**A**); Catalase (**B**) and superoxide dismutase (SOD) (**C**) activity; Malondialdehyde (MDA) (**D**), Nitrite (**E**) and reactive oxygen species (ROS) levels (**F**). *** *p* < 0.001 vs. control, ## *p* < 0.01 vs. CLP, ### *p* < 0.001 vs. CLP.

*Coriolus versicolor* administration restored the GSH levels (Figure 12A) and catalase (Figure 12B) and SOD (Figure 12C) activity, which were reduced by CLP. CLP also increased lipid peroxidation (Figure 12D), nitrite (Figure 12E), and ROS levels (Figure 12F), as compared to the controls, which were decreased in the CLP + *Coriolus versicolor* group. No statistical differences were detected between Control and ontrol + *Coriolus versicolor* groups. 

### 3.9. Effects of Coriolus versicolor on AD-like Neuropathology CLP Induced

**Figure 13:**
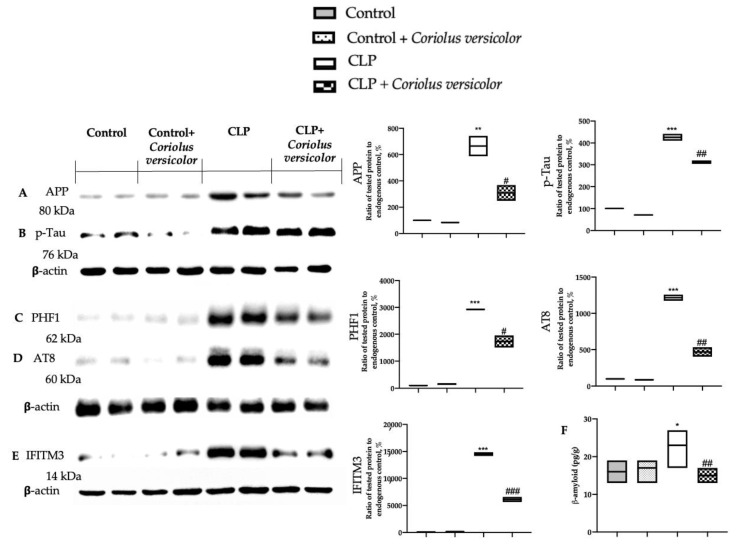
*Coriolus versicolor* reduced AD-like neuropathology induced by CLP 28 days after surgery. Western blot analysis of amyloid precursor protein (APP) (**A**), phosphorylated-Tau (p-Tau) (**B**), pathologically phosphorylated tau (PHF1) (**C**), phosphorylated tau (Ser202 and Thr205) (AT8) (**D**), and interferon-induced transmembrane protein 3 (IFITM3) (**E**) expression; β-amyloid levels (**F**). * *p* < 0.05 vs. control, # *p* < 0.05 vs. CLP, ** *p* < 0.01 vs. control, ## *p* < 0.01 vs. CLP, *** *p* < 0.001 vs. control, ### *p* < 0.001 vs. CLP.

**Figure 14:**
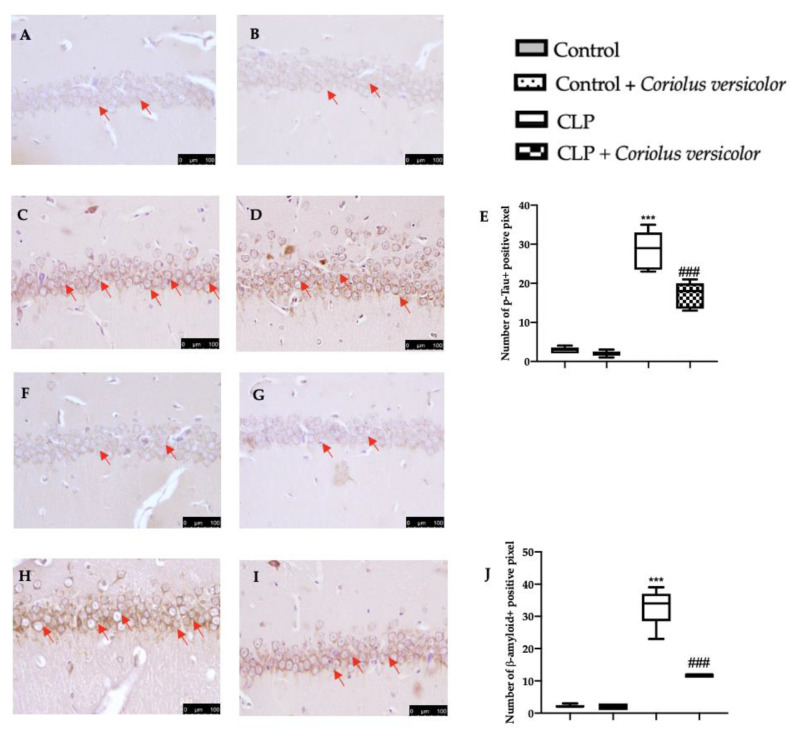
*Coriolus versicolor* reduced phosphorylated-Tau (p-Tau) and Aβ expression in hippocampus induced by CLP 28 days after surgery. Immunohistochemical analysis of p-Tau: Control (**A**), Control + *Coriolus versicolor* (**B**), CLP (**C**), CLP + *Coriolus versicolor* (**D**), graphical quantification of p-Tau expression (**E**), immunohistochemical analysis of β-amyloid: Control (**F**), Control + *Coriolus versicolor* (**G**), CLP (**H**), CLP + *Coriolus versicolor* (**I**), graphical quantification of β-amyloid expression (**J**). *** *p* < 0.001 vs. control, ### *p* < 0.001 vs. CLP.

To evaluate the effect of administration on *Coriolus versicolor* AD-like pathology, Western blot analysis was conducted. Increased amyloid precursor protein (APP) (Figure 13A), phosphorylated-Tau (p-Tau) (Figure 13B), pathologically phosphorylated tau (PHF1) (Figure 13C), phosphorylated tau (Ser202 and Thr205) (AT8) (Figure 13D), and interferon-induced transmembrane protein 3 (IFITM3) (Figure 13E) expression was detected in tissues harvested from the CLP group, as compared to the Control and Control + *Coriolus versicolor* groups. *Coriolus versicolor* administration significantly decreased these levels and also reduced β-amyloid accumulation (Figure 13F), as compared to CLP. Immunohistochemical analysis confirmed the increased expression of p-Tau and β-amyloid in the CLP group (Figure 14C, Figure 14D, Figure 14H, and Figure 14J, respectively), as compared to the Control (Figure 14A,F) and Control + *Coriolus versicolor* groups (Figure 14B,G). Well in line with the Western blot results, the CLP + *Coriolus versicolor* group showed reduced p-Tau (Figure 14D) and β-amyloid (Figure 14I) expression, as compared to CLP.

### 3.10. Effects of Coriolus versicolor on Behavioral Alteration CLP Induced

Behavioral analyses were performed to evaluate the modification induced by CLP and the effects of *Coriolus versicolor* treatment.

**Figure 15:**
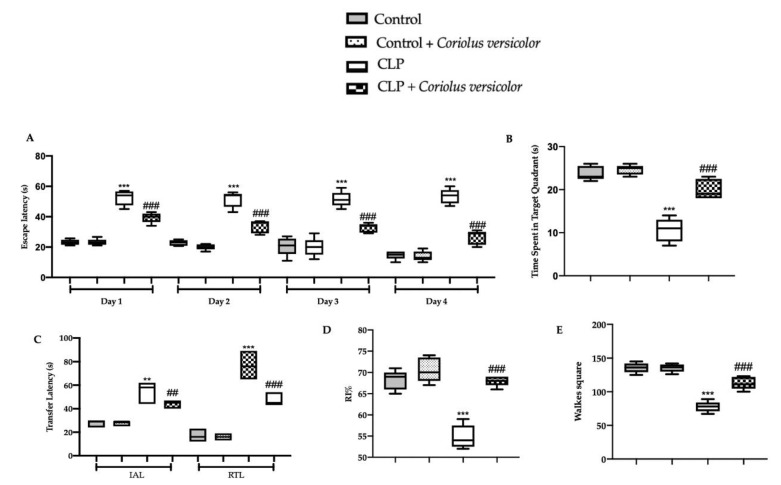
*Coriolus versicolor* reduced behavioral alterations induced by CLP 28 days after surgery. Morris water maze test: training (**A**), probe trial (**B**); Elevated plus maze test (**C**); Novel object recognition test (**D**); Open field test (**E**). ** *p* < 0.01 vs. control, ## *p* < 0.01 vs. CLP, *** *p* < 0.001 vs. control, ### *p* < 0.001 vs. CLP.

In the training test of the MWM test, on day four the animals from all the groups displayed a decreasing trend in the escape latency time as compared to that on day one (Figure 15A). In the probe trial, *Coriolus versicolor* administration increased the time spent by the animals in the target quadrant, demonstrating an increase in memory consolidation as compared to the CLP group (Figure 15B). In the EPM test, the CLP + *Coriolus versicolor* group showed a reduced time of travel of the animal from the open arm to the closed arm (initial acquisition latency, IAL) and reduced retention of memory (RTL), demonstrating an increase in memory retention as compared to the CLP group (Figure 15C). In the NOR test, *Coriolus versicolor* administration significantly increased the RI%, demonstrating an increase in cognitive function, which was reduced in the CLP group (Figure 15D). No statistical differences were detected between Control and Control + *Coriolus versicolor* groups. The locomotion and additional probing behaviors of rats were inspected via the open field test. A diminution in the locomotion was observed in the CLP group, with an augmented time that was spent motionless while comparing it to the controls (Figure 15E). Interestingly, the *Coriolus versicolor* administration revealed a noticeable enhancement in the locomotion and substantial reduction in the time spent motionless phase, as compared to the CLP group. Additionally, there were no variations noted between the control and the control + *Coriolus versicolor* groups. 

### 3.11. Effects of Coriolus versicolor on Synaptic Plasticity CLP Induced

**Figure 16:**
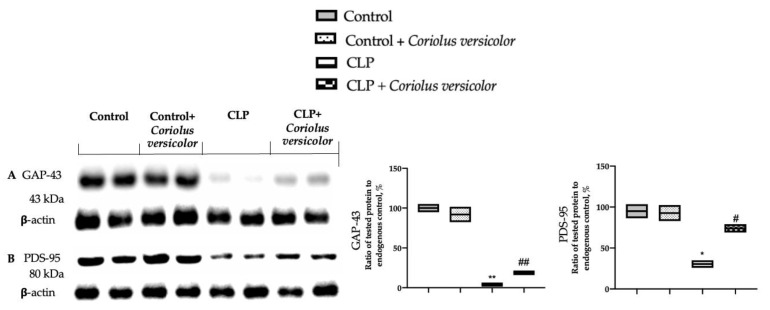
*Coriolus versicolor* restored synaptic injury in hippocampus induced by CLP 28 days after surgery. Western blot analysis of growth-associated protein-43 (GAP-43) (**A**) and postsynaptic density protein 95 (PSD-95) (**B**) expression. A *p*-value of less than 0.05 was considered significant. * *p* < 0.05 vs. control, # *p* < 0.05 vs. CLP, ## *p* < 0.01 vs. CLP, ** *p* < 0.01 vs. control.

The expressions of growth-associated protein-43 (GAP-43) and postsynaptic density protein 95 (PSD-95) were assessed to investigate the synaptic plasticity. Western blot analysis showed decreased expression of GAP-43 (Figure 16A) and PSD-95 (Figure 16B) in the CLP group, as compared to Control and Control + Coriolus versicolor groups. *Coriolus versicolor* administration restored levels of GAP-43 and PSD-95, as compared to CLP.

## 4. Discussion

Numerous studies have examined the acute inflammatory response to sepsis, modeled either by CLP or high dose LPS in rodent models. In this study, *Coriolus versicolor* administration improved the survival and tissue injury induced by CLP in the acute phase of sepsis. Indeed, rats surviving sepsis showed brain degeneration strongly associated with the onset of Alzheimer disease. Here we demonstrated the effect of *Coriolus versicolor* administration in the relationship between the enhanced transcription of the pro-inflammatory cytokines and increased BBB permeability, which induces hippocampal plasticity, neuroinflammation, and impaired cognitive functions [28,50]. 

We acknowledge that our study has potential limitations. First, we did not perform the same analysis in the “100%” group because of the high lethality rate. We chose animals with the same genetic background, age, and gender to limit experimental variability secondary to differences in inflammatory response and maturity of the immune system [51,52]. Furthermore, our study lacked evaluation of anti-inflammatory balance within the first hours post-procedure. We were not able to determine if the 100% group had a very early pro- and anti-inflammatory imbalance, which has been shown to be predictive of mortality [53]. Third, we are aware that we have not exhaustively analyzed the cytokine response to cecal ligation. For example, other cytokines, such as IL-12 or interferon-γ, play a central role in septic inflammatory response [54].

The dose of *Coriolus versicolor* was chosen basing on previous studies where it already displayed neuroprotective effects [23]. Sepsis induced a strong release of pro-inflammatory cytokines in the bloodstream that can induce inflammation and injury. *Coriolus versicolor*, with its antimicrobial, anti-inflammatory, and antioxidant activities, increased survival and reduced the release of pro-inflammatory cytokines in the bloodstream, leading to reduced systemic inflammation. Previous studies associated the systemic inflammation to the sepsis-induced BBB dysfunction [55]. The BBB is a biochemical and structural barrier that regulates the access of molecules from the plasma to the brain, preserving the CNS homeostasis. It is constituted by microvascular endothelial cells that are closely linked by tight junctions [56,57]. These tight junctions are the functionally important part of the barrier because they modulate its function [58,59]. Previous papers have shown that decreasing the loss of occludin and ZO-1 proteins could restore the BBB permeability [60,61]. The CLP-induced BBB impairment was importantly restored by *Coriolus versicolor* administration, as shown by the increased expression of ZO-1 and occludin. Once the BBB is damaged, cytokines and proinflammatory mediators reach the brain and induce neuroinflammation. Brain proinflammatory markers increased in animals subjected to CLP [62]. Previous papers have demonstrated the relationship between CLP and microglial activation and the related long-cognitive disfunctions [16]. *Coriolus versicolor* administration was able to reduce cytokines accumulation in the brain and GFAP and Iba-1 expression, which is a well-known marker of astrocytes and microglia. The glia activation has an important role in chronic neuroinflammation. In this context, the activation of the NLRP3 inflammasome complex and TLR4 and nNOS overexpression have been related to astrocytes/microglia activation [63]. In particular, TLR4 activates the production of nitric oxide (NO), cytokines, and ROS in CNS. *Coriolus versicolor* administration by reducing glia stimulation strongly reduced the expression of the NLRP3 inflammasome complex components, TLR4 and nNOS. *Coriolus versicolor* also has important antioxidant activities: it reduced lipid peroxidation, nitrite and ROS levels, and restored the activities of the phase II detoxifying enzymes, including SOD, Catalase, and GSH. In sequence, the pro-inflammatory environment stimulates the formation of IFITM3 protein in astrocytes and neurones [64], which upregulate Aβ production. Its accumulation in amyloid plaques is regulated by transcriptional directive of AβPP, modification in activity, and/or expression of the secretases involved in AβPP cleavage and ROS expression resulting from glial activation [65]. The Aβ deposition further increased the cerebral inflammatory answer. IFITM3 is a γ-secretase modulatory protein, which is basally expressed in many cells and found upregulated in AD patients [64]. Increased Tau phosphorylation is correlated with numerous neurodegenerative disorders [66]. The involvement of abnormal Tau phosphorylation in brain impairment following sepsis is in line with previous studies, including the decline of cognitive function and oxidative stress to CNS [67,68]. Our works revealed that *Coriolus versicolor* administration by reducing neuroinflammation decreased the expression of IFITM3, APP protein, Aβ deposition, Tau phosphorylation, and PHA1 and AT8 expression. 

These molecular events are often associated with behavioral AD-like modifications, including impairment in memory retention tasks and spontaneous locomotor activities. Well in line with all these results, *Coriolus versicolor* administration ameliorated the cognitive impairment associated with sepsis-induced neuroinflammation. Cognitive impairments are often associated with synaptic degeneration [69]. Biomarkers for synaptic dysfunction can be divided into pre- and postsynaptic biomarkers depending on the localization of the protein [70]. The protein GAP-43, found in high levels in the prefrontal cortex and hippocampus, plays a crucial role in axonal growth, regeneration, and synaptogenesis [71]. When GAP-43 is phosphorylated, it encourages the growth of neurites and boosts vesicle cycling, which leads to heightened neuronal plasticity [72]. Furthermore, PSD-95 is a postsynaptic scaffolding protein that participates in the development and malleability of synapses [73]. Consistent with prior findings, treatment with *Coriolus versicolor* boosts the production of GAP-43, a presynaptic protein, and PSD-95, a postsynaptic protein, in the hippocampus.

## 5. Conclusions

Overall, this paper demonstrated that *Coriolus versicolor* administration was able to counteract the acute phase of sepsis and the chronic the neurodegenerative processes. Importantly, *Coriolus versicolor* reduced microglia and astroglia activation, Aβ deposition, and the related long-cognitive disfunctions.

## Figures and Tables

**Table 1:** Primary antibodies.

Antibody	Product Name
Anti-TLR4	Santa Cruz Biotechnology, sc-293072, Milan, Italy
Anti-nNOS	Invitrogen, Milan, Italy
Anti-IFITM3	Santa Cruz Biotechnology, sc-100768, Milan, Italy
Anti-NLRP3	Cell Signaling Technology, Danvers, MA, USA
Anti-ASC	Santa Cruz Biotechnology, sc-271054, Milan Italy
Anti-Caspase-1	Cell Signaling Technology, Danvers, MA, USA
Anti-p-Tau	Santa Cruz Biotechnology, sc-32275, Milan, Italy
Anti-APP	Santa Cruz Biotechnology, sc-32277, Milan, Italy
Anti-occludin	Santa Cruz Biotechnology, sc-133255, Milan, Italy
Anti-ZO	Santa Cruz Biotechnology, sc-33725, Milan, Italy
Anti-AT8	Invitrogen MN1020, Milan, Italy
Anti-PHF1	Santa Cruz Biotechnology, sc-515013, Milan, Italy
Anti-GAP-43	Santa Cruz Biotechnology, sc-33705, Milan, Italy
Anti-PSD-95	Abcam, ab2723, Milan, Italy
Anti-GFAP	Santa Cruz Biotechnology, sc-33673, Milan, Italy
Anti-Iba-1	Santa Cruz Biotechnology, sc-32725, Milan, Italy
Anti-β-amyloid	Sigma AB5078P, Milan, Italy

## Data Availability

The data that support the findings of this study are available in the methods of this article. The rest of the data will be available from the corresponding author on reasonable request.
